# Summer Drinks

**Published:** 1870-07

**Authors:** 


### SUMMER DRINKS.

No beers, or wines, or brandies — nothing alcoholic! They are all carbonaceous, and increase the heat and fever of the system; they clog up, instead of lighten; they debilitate instead of giving strength. The tendency of the system in all lands in summer is to biliousness — to bilious diseases; we call them fevers. This is because we eat more than we wear out or work out by exercise or labor; hence, the body becomes too full. The great scavenger or emptier of the body is the liver; it is like the pump in a sinking vessel: you must keep it at work, or all is lost. As warm weather renders us incapable of doing as much work in summer as in winter, and as work was intended, in part, to work out the wastes of the system, some substitute must be provided, some compensating power. That power is the liver. It must be made to do more work in summer; and a beneficent Providence has made provision for this in sending us the berries and fruits of warm weather, which contain an ingredient, the acid, which, after six thousand years, the French found out has its efficacy towards cooling off the body by acting on the liver, — having an effect upon it to make it work more actively, and thus more promptly remove the bile, which is the waste matter of the system, from the blood. Everybody knew that fruits and berries were cooling, were " healthy ; " but how they were, was not definitely known until within a very few years. The wisdom and benevolence of our Maker in this thing, surely will command our affections, in that he has provided these fruits and berries in such generous profusion and combined a necessary quality with such a delicious taste, that every soul of man is perfectly ravenous for them, and we can eat them, without harm, to our utmost fill, if ripe, raw, and perfect, and taken alone.

In the absence of fruits and berries, we may obtain the needed natural acid from the lemon, diluted with water, but the most universally available acid drink for summer, as a natural aid to the liver, is buttermilk, not an ounce of which should ever be wasted, to be taken at meal-times, or between meals when thirsty.

FOR THE HARVEST FIELD, buttermilk is the safest, most healthful and cooling of summer drinks, to be taken at the temperature of the air; if a mouthful is swallowed at a time, with a distinct interval, the thirst will be better satisfied with a quarter of a pint, than if a whole cupful is taken without being removed from the lips.

A palatable and safe summer drink for out-door workers, is water of the natural temperature, sweetened with molasses. All root beers are pernicious, for being without appreciable nutriment, they cannot add to the strength of the body, and their value is deceptive.

